# Oxidative stress induces the acquisition of cancer stem-like phenotype in breast cancer detectable by using a Sox2 regulatory region-2 (SRR2) reporter

**DOI:** 10.18632/oncotarget.6630

**Published:** 2015-12-16

**Authors:** Keshav Gopal, Nidhi Gupta, Haifeng Zhang, Abdulraheem Alshareef, Hind Alqahtani, Gilbert Bigras, Jamie Lewis, Donna Douglas, Norman Kneteman, Afsaneh Lavasanifar, Raymond Lai

**Affiliations:** ^1^ Department of Laboratory Medicine and Pathology, University of Alberta, Edmonton, Alberta, Canada; ^2^ Department of Surgery, University of Alberta, Edmonton, Alberta, Canada; ^3^ Faculty of Pharmacy and Pharmaceutical Sciences, University of Alberta, Edmonton, Alberta, Canada; ^4^ Department of Chemical and Materials Engineering, University of Alberta, Edmonton, Alberta, Canada; ^5^ Department of Oncology, University of Alberta, Edmonton, Alberta, Canada

**Keywords:** breast cancer, Sox2, H_2_O_2_, acquisition of stemness, plasticity

## Abstract

We have previously identified a novel intra-tumoral dichotomy in breast cancer based on the differential responsiveness to a Sox2 reporter (SRR2), with cells responsive to SRR2 (RR) being more stem-like than unresponsive cells (RU). Here, we report that RR cells derived from MCF7 and ZR751 displayed a higher tolerance to oxidative stress than their RU counterparts, supporting the concept that the RR phenotype correlates with cancer stemness. Sox2 is directly implicated in this differential H_2_O_2_ tolerance, since siRNA knockdown of Sox2 in RR cells leveled this difference. Interestingly, H_2_O_2_ converted a proportion of RU cells into RR cells, as evidenced by their expression of *luciferase* and *GFP*, markers of SRR2 activity. Compared to RU cells, converted RR cells showed a significant increase in mammosphere formation and tolerance to H_2_O_2_. Converted RR cells also adopted the biochemical features of RR cells, as evidenced by their substantial increase in Sox2-SRR2 binding and the expression of 3 signature genes of RR cells (*CD133, GPR49 and MUC15*). Lastly, the H_2_O_2_-induced RU/RR conversion was detectable in a SCID mouse xenograft model and primary tumor cells. To conclude, the H_2_O_2_-induced RU/RR conversion has provided a novel model to study the acquisition of cancer stemness and plasticity.

## INTRODUCTION

Similar to many other types of solid tumors, breast cancer (BC) contains a small subset of cells carrying stem-like features, which are commonly labeled cancer stem cells (CSCs) [1]. In triple-negative BC, CSCs are enriched in the CD44^high^/CD24^low^ subpopulation [2]. In these tumors, CSCs have been shown to contribute to metastasis, chemoresistance and a worse clinical outcome [3-5]. In estrogen receptor-positive BC, the identity of the CSCs is more controversial. While a few studies have shown that the CD44^high^/CD24^low^ is also a marker of CSCs in this subtype of BC, other studies did not find the association between this immunophenotype and cancer stemness [6, 7]. In recent years, the biology of CSCs has become an active area of research, with the hope that the combination of conventional chemotherapy with specific CSC inhibitors can dramatically improve the clinical outcome of BC patients [8]. One of the important features of CSCs is plasticity. Specifically, a few recent studies have demonstrated that the status of CSCs can be acquired [9-11]. Thus, mammary epithelial cells can be de-differentiated into stem-like cells upon telomerase transfection [12]. Cancer stemness also can be acquired in response to adversity such as oxidative stress. In one study, hypoxia, which is a cause of oxidative stress, was found to increase the expression of stem cell-associated genes and tumorigenic potential in non-stem cells [13]. In another study, it was found that hypoxia results in the acquisition of stemness in glioma cells [14]. Likewise, oxidative stress induced by H_2_O_2_ has been shown to enhance the stem-like properties in human mesothelioma cells and primary brain-derived neural progenitors cells [15, 16]. Lastly, hypoxia in conjunction with H_2_O_2_ has been reported to enhance tumor stemness by increasing the fractions of side cell population, which is highly migratory, invasive, and tumorigenic in a variety of solid tumor cell lines [17].

Sox2 (sex determining region Y-box protein 2) is a transcription factor that plays a major role in maintaining the pluripotency of embryonic stem cells and induced pluripotent stem (iPS) cells [18, 19]. Recent studies have shown that Sox2 is aberrantly expressed in several types of solid tumors including breast cancer, lung cancer, prostate cancer, glioblastomas and melanomas [20-24]. Furthermore, Sox2 has been found to correlate with a worse prognosis in cancer patients, including those with BC [25-27]. Our previous studies using a Sox2 reporter construct (SRR2) have revealed a novel dichotomy in BC cells [28]. Specifically, using two estrogen receptor-positive BC cell lines, MCF7 and ZR751, we have found that the vast majority of these cells were reporter unresponsive (RU), despite the fact that these cells strongly expressed the Sox2 protein. In contrast, a relatively small cell subset were reporter responsive (RR), and RR cells were more stem-like than RU cells in terms of phenotype and gene expression patterns [28, 29]. Nonetheless, the relationship between RU and RR cells needs to be further defined. For instance, it will be of interest to determine if RU cells can be induced to acquire the phenotype of RR cells, analogous to the above-mentioned examples in which non-stem cells were found to acquire cancer stemness.

Our first study objective is to assess if RR cells are more tolerant to oxidative stress, a phenotype known to be associated with cancer stemness, than RU cells. During the course of our studies, we also found that RU cells can be induced in response to oxidative stress to acquire SRR2 responsiveness (i.e. to become converted RR cells). In view of these findings, the second objective of this study is to understand the nature of this RU/RR conversion, by performing comparative studies of RU, converted RR cells and native RR cells. This study not only has provided additional support of the biological significance of the RU/RR dichotomy in BC, it also has generated a novel experimental model to study cancer cell plasticity.

## RESULTS

### RR cells are more tolerant to oxidative stress than RU cells

**Figure 1:**
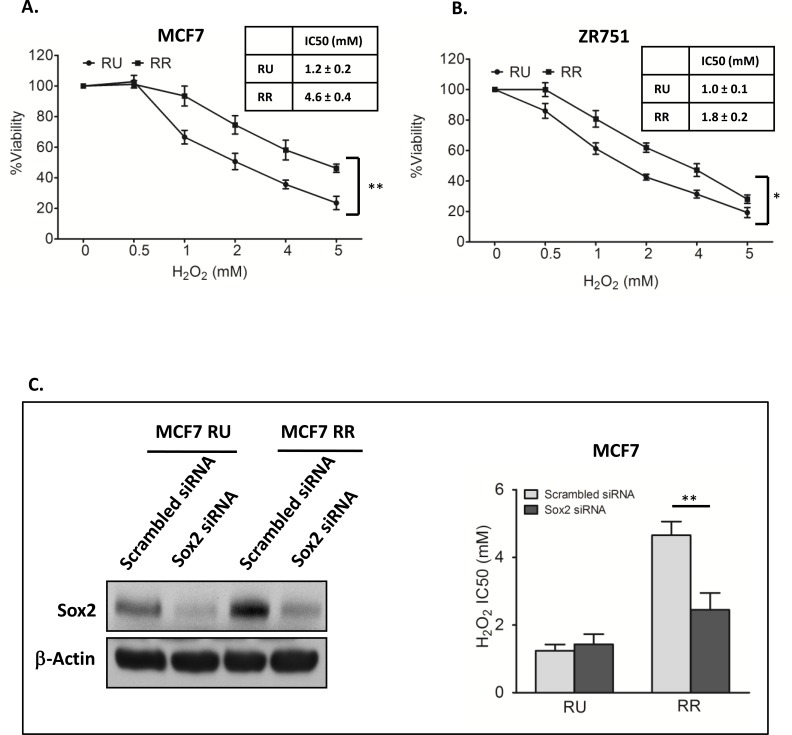
Sox2 activity increased H_2_O_2_ resistance in BC cells **A**. RU and RR cells derived from MCF7 were exposed to varying doses of H_2_O_2_ for 2 hours in serum free media. MTS assay was used to assess the cell viability at the end of the experiments. Data is expressed as percentages of the negative control cells, which were set as 100%. RR cells were significantly more resistant than RU cells (4.6 mM versus 1.2 mM, p<0.01). **B**. The same experiment was repeated using ZR751, which showed similar results (1.8 mM versus 1.0 mM, p<0.05). **C**. RU and RR cells derived from MCF7 cells were transfected with *Sox2* siRNA for 48 hours, western blots was done to confirm the knockdown efficiency, as compared to the scrambled siRNA negative control. β-actin serves as a loading control (left panel). These cells were then exposed to varying doses of H_2_O_2_ for 2 hours in serum free media. Knockdown of Sox2 significantly decreased the IC50 of RR cells, which was at a level similar to that of RU cells.

We first asked if RR cells differ from RU cells with regards to their sensitivity to oxidative stress. We subjected RR and RU cells derived from MCF7 to varying concentrations of H_2_O_2_ in serum free media. As shown in Figure 1A, the number of viable cells at 2 hours, assessed by using the MTS assay, decreased in a dose-dependent manner in both RR and RU cells, although the IC50 (inhibitory concentration at 50%) was significantly higher in RR cells as compared to RU cells (4.6 mM versus 1.2 mM, *p* < 0.01). The same phenomenon was observed when we used RR and RU cells derived from ZR751, another estrogen receptor-positive BC cell line (Figure 1B).

### Sox2 directly contributes to the high tolerance to oxidative stress in BC cells

As we have previously shown that siRNA knockdown of Sox2 can abrogate the SRR2 reporter activity in RR cells derived from MCF7 [28], we asked if siRNA knockdown of Sox2 can result in any significant change to their tolerance to H_2_O_2_. As shown in Figure 1C, siRNA significantly decreased the IC50 of RR cells in response to H_2_O_2_, to a level similar to that of RU cells. In comparison, siRNA knockdown of Sox2 did not significantly change the IC50 of RU cells. Thus, Sox2 is directly responsible for the relative high tolerance to oxidative stress in RR cells.

### Oxidative stress can induce a conversion of RU cells to RR cells

**Figure 2:**
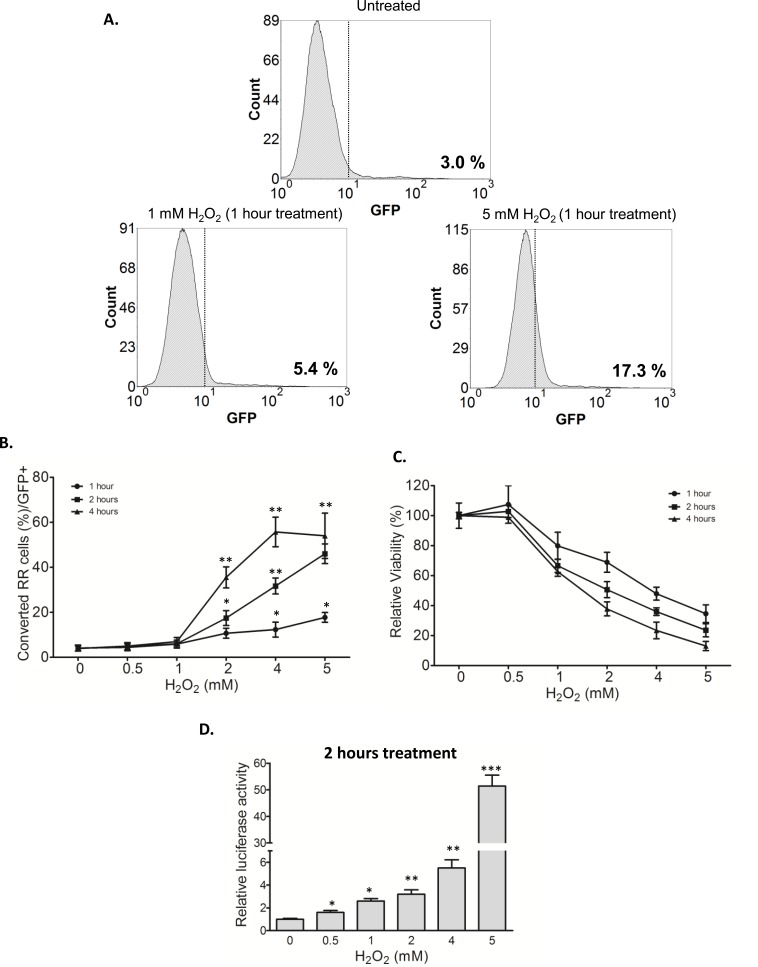
RU cells converted to RR cells upon H_2_O_2_ challenge **A**. RU cells derived from MCF7 were exposed to varying doses of H_2_O_2_ for 1 hour in serum free media. Flow cytometry was used to assess the expression of GFP in the viable cell populations. Data is expressed relative to untreated negative control cells and the values represent the GFP positive cells. Addition of H_2_O_2_ to RU cells increased the proportion of GFP-positive cells (from 3.0%, background level to 17.3%). **B**. Data is expressed as percent of cells with higher GFP expression relative to untreated negative control detected by flow cytometry (called converted RR cells/GFP+) after exposure to varying doses of H_2_O_2_ for different time points in serum free media. The proportions of converted RR cells (or GFP-positive) significantly increased in a time- and dose-dependent fashion. **C**. Cells from above experiments were subjected to MTS assay to assess the cell viability at the end of experiments. Data is expressed as percentages of the negative control cells, which were set as 100%. The cell viability decreased in a time- and dose-dependent fashion. **D**. RU cells derived from MCF7 were exposed to varying doses of H_2_O_2_ for 2 hour in serum free media. Data is expressed as luciferase activity relative to untreated negative control. RU cells treated with H_2_O_2_ significantly increased the luciferase activity in a dose-dependent manner.

Our previous studies have suggested that RR cells derived from MCF7 and ZR751 have more stem-like features and tumorigenicity than their RU counterparts [28]. Furthermore, previous studies have shown that cancer stemness can be acquired in response to oxidative stress [15-17]. Thus, we asked if oxidative stress can convert RU to RR cells, a phenomenon that might represent the acquisition of cancer stemness and exemplify the concept of cancer cell plasticity. We tested this possibility by using purified RU cells derived from MCF7. As illustrated in Figure 2A, addition of H_2_O_2_ to RU cells increased the proportion of GFP-positive cells (i.e. a surrogate marker of the RR phenotype) as early as 1 hour. Specifically, 1 mM of H_2_O_2_ increased the GFP-positive cells from 3.0% (background level) to 5.4% whereas 5 mM of H_2_O_2_ increased to 17.3%. As shown in Figure 2B, the proportions of converted RR cells (or GFP-positive) significantly increased in a time- and dose-dependent fashion. Details of the flow cytometry study results are included in Supplemental Figure 1A. In the same experiment, the cell viability also decreased in a time- and dose-dependent fashion (Figure 2C).

To ensure that the expression of GFP induced by H_2_O_2_ was genuine, we assessed if the converted RR cells also express luciferase, another readout marker included in the SRR2 reporter. As shown in Figure 2D, the luciferase activity in RU cells treated with H_2_O_2_ significantly increased in a dose-dependent manner. To highlight the magnitude of this biological change, RU cells treated with 5 mM H_2_O_2_ for 2 hours showed a 51-fold increase as compared to the negative control. No substantial change in the GFP expression or luciferase activity was observed in RR cells treated with H_2_O_2_ (Supplemental Figure 1A and 1B).

**Figure 3:**
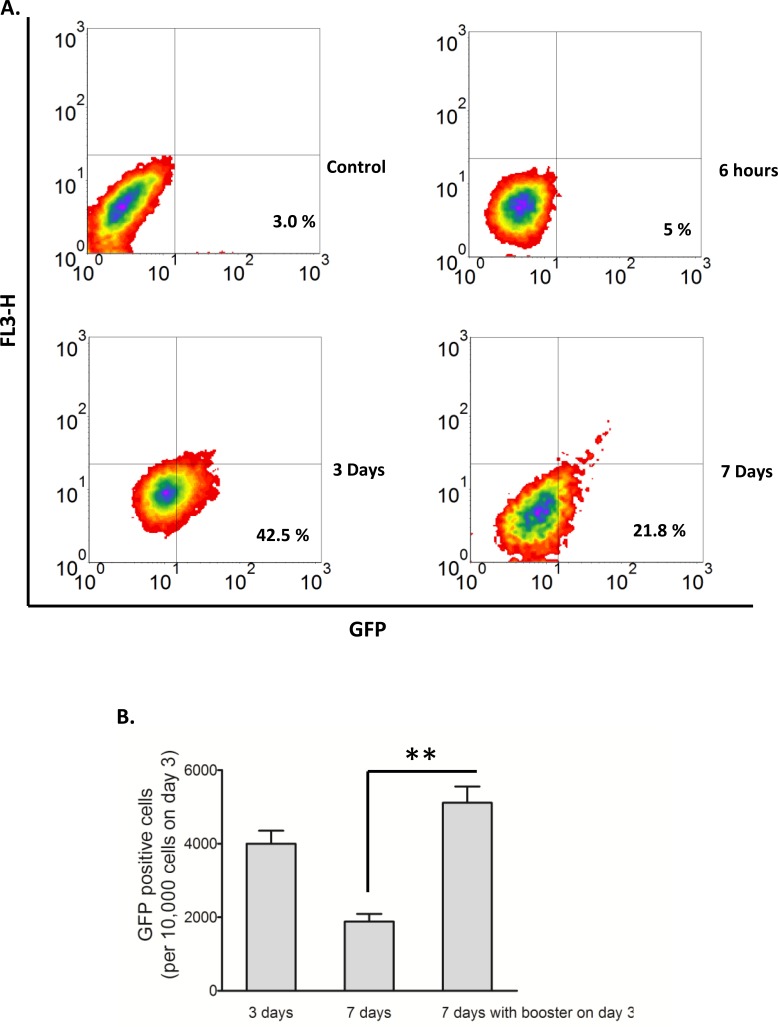
Conversion of RU to RR stayed longer with modified culture conditions **A**. RU cells derived from MCF7 were exposed to 0.5 mM H_2_O_2_ for 6 hours in culture media supplemented with 10% FBS and further cultured in fresh media. Flow cytometry was used to assess the expression of GFP in the viable cell populations. Data is expressed relative to untreated negative control cells and the values represent the GFP positive cells. Addition of H_2_O_2_ to RU cells increased the proportion of GFP-positive cells from 3.0%, background level to 42.5% on day 3, which later decreased to 21.8% on day 7. **B**. The converted RR cells on day 3 of above experiment without separating them from RU cells were re-exposed to 0.5 mM H_2_O_2_ for 6 hours and further cultured in fresh media. Data were plotted as number of converted RR cells normalized to 10,000 cells at the beginning of re-exposure. With the addition of a ‘booster’ H_2_O_2_ treatment (0.5 mM) on day 3, the number of viable converted RR cells was significantly increased on day 7 (5000 cells versus 2000 cells, *p* < 0.01).

To optimize the yield of viable H_2_O_2_-induced converted RR cells, we attempted different H_2_O_2_ treatment protocols. Our optimized protocol involved treatment of RU cells with 0.5 mM H_2_O_2_ in complete growth media for 6 hours, followed by washing and a 3-day routine culture (also see Methods and Materials). The yield was consistently in the approximate of 4,000 viable converted RR cells per 10,000 RU cells used at the beginning of the experiments. If these cells were cultured for another 4 days (or 7 days in total), the number of viable converted RR cells was found to decrease to approximately 2,000 cells, probably due to the expansion of the RU cells and/or a conversion back to RU cells. These results are illustrated in Figure 3A. With the addition of a ‘booster’ H_2_O_2_ treatment (0.5 mM) on day 3, the number of viable converted RR cells was significantly increased on day 7; an approximate of 5,000 cells for every 10,000 RU cells used at the beginning of the experiment was obtained (Figure 3B). Using these protocols, we obtained sufficient numbers of viable converted RR cells for further characterization, to be detailed below.

### H_2_O_2_-induced RR conversion is dependent on the anti-oxidant scavenger pathway

**Figure 4:**
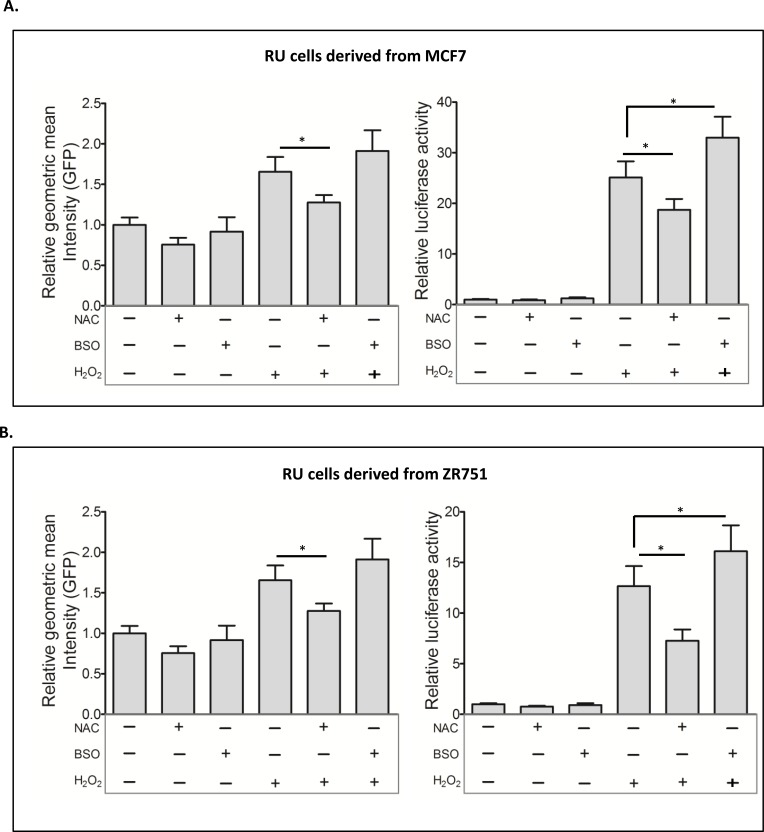
Glutathione modulations affected the RU to RR conversion **A**. RU cells derived from MCF7 were exposed to NAC and BSO prior to H_2_O_2_ treatment. Flow cytometry was used to assess the expression of GFP in the viable cell populations. Data is expressed as mean ± standard error of geometric mean intensity of GFP relative to untreated negative control. NAC pretreatment significantly reduced the H_2_O_2_ induced GFP expression (2.5 versus 1.7, *p* < 0.05) (left panel). The cells from same experiments were subjected to luciferase assay. Data is expressed as luciferase activity relative to untreated negative control. NAC and BSO pre-treatment significantly attenuated (18.7 versus 25.1, *p* < 0.05) and enhanced (32.9 versus 25.1, *p* < 0.05) the H_2_O_2_-induced luciferase activity, respectively (right panel). **B**. The same experiment was repeated using RU cells derived from ZR751, which showed similar results.

To test that the RR conversion induced by H_2_O_2_ is directly related to the cellular response to oxidative stress, we experimentally manipulated the anti-oxidant scavenger pathway using N-acetyl-L-cysteine (NAC) and buthionine-sulfoximine (BSO), which can increase or deplete glutathione, respectively [30-32]. As shown in Figure 4A, NAC pretreatment of RU cells derived from MCF7 significantly attenuated H_2_O_2_-induced conversion to RR. In comparison, BSO pretreatment increased the RR conversion, as evidenced by the increases in GFP expression (*p* = 0.07) and luciferase (*p* < 0.05). Similar results were obtained with RU cells derived from ZR751 (Figure 4B). The lack of a statistical significance in the change in GFP is likely due to the fact that the GFP read-out is not as sensitive as luciferase.

### Converted RR cells are phenotypically similar to native RR cells

**Figure 5:**
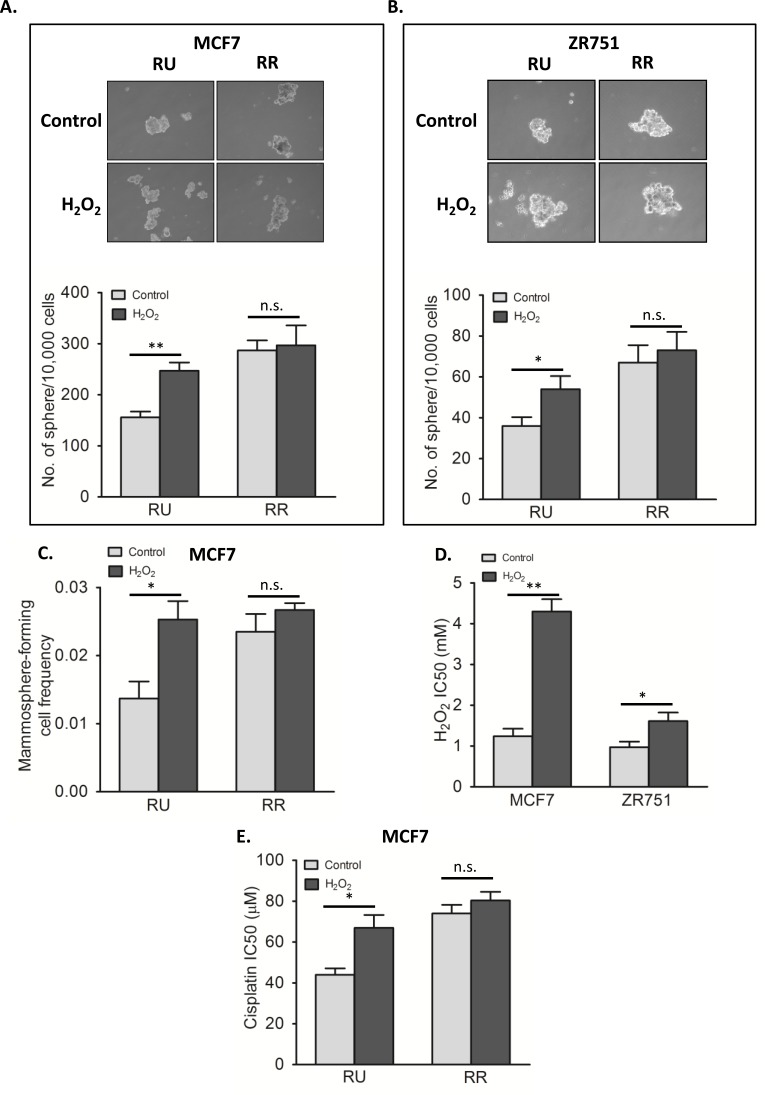
Converted RR cells acquired phenotypes of RR cells **A**. RU and RR cells derived from MCF7 were exposed to 0.5 mM H_2_O_2_ for 6 hours in complete culture media and further cultured for 3 days in fresh media. The harvested cells were subjected to mammosphere formation assay. Data is expressed as mean ± standard error of number of spheres per 10000 cells. RU cells upon H_2_O_2_ treatment (converted RR cells) showed significantly higher number of sphere as compared to untreated control (247 versus 156 sphere, *p* < 0.01). RR cells did not show any significant change in sphere formation upon H_2_O_2_ treatment. The representative mammosphere pictures are shown (upper panel). **B**. The same experiment was repeated using RU and RR cells derived from ZR751, which showed similar results (54 versus 36 sphere, *p* < 0.05 in case of RU cells) **C**. RU and RR cells derived from MCF7 were exposed to 0.5 mM H_2_O_2_ for 6 hours in complete culture media and further cultured for 3 days in fresh media. The harvested cells were seeded in 10 seeding densities ranging from 1 to 400 cells/well in 96-well plate in 6 replicates each. Data is expressed as mammosphere-forming cell frequency obtained by using extreme limiting dilution analyses. RU cells upon H_2_O_2_ treatment (converted RR cells) showed significant increase in mammosphere formation frequency (0.025 versus 0.014, *p* < 0.05). RR cells did not show any significant change. **D**. Converted RR cells and RU cells derived from MCF7 and ZR751 were exposed to varying doses of H_2_O_2_ for 2 hours with serum free media followed by assessment of cell viability by MTS assay. Data is expressed as mean ± standard errors of IC50 values calculated by using Graphpad Prism software. Converted RR cells were significantly more resistant than RU cells (in-case of MCF7, 4.3 mM versus 1.2 mM, *p* < 0.01; in-case of ZR751, 1.6 mM versus 1.0 mM, *p* < 0.05). **E**. The same experiment was performed with varying doses of cisplatin for 24 hours with RU and RR cells derived from MCF7, which showed similar results. Converted RR cells were significantly more resistant to cisplatin than RU cells (66.9 μM versus 44 μM, *p* < 0.05). RR cells did show any significant change upon H_2_O_2_ exposure.

As mentioned, we have previously shown that RR cells are more efficient in forming mammospheres compared to RU cells, a finding that is in keeping with their higher stem-like properties [28]. To evaluate the biological significance of the H_2_O_2_-induced RR conversion, we tested if converted RR cells are phenotypically similar to the native RR cells with regards to mammosphere formation. As shown in Figure 5A, converted RR cells derived from MCF7 harvested on day 3 were subjected to mammosphere formation assay, and they formed a significantly higher number of mammospheres than untreated cells (*p* < 0.01). Of note, since only approximately 2/3 of all viable cells on day 3 were converted RR cells (i.e. 1/3 was viable RU cells), the actual increase in the mammosphere formation ability of converted RR cells probably is substantially higher than the observed changes. Similar results were obtained with ZR751 cells (Figure 5B).

To further assess the stemness of converted RR cells, we performed the limiting dilution assay, which has been widely used to quantify the number of cancer stem-like cells. [33, 34]. As shown in Figure 5C, converted RR cells had a mammosphere-forming cell frequency of 0.025, which is significantly higher than that of native RU cells (0.014, *p* < 0.05).

We then asked if converted RR cells acquired the resistance towards H_2_O_2_. As shown in Figure 5D, converted RR cells from both MCF7 and ZR751 showed significantly higher IC50 value as compared to untreated RU cells (MCF7; 4.3 mM versus 1.2 mM, *p* < 0.01, and ZR751; 1.6 mM versus 1.0 mM, *p* < 0.05). We also evaluated the effectiveness of cisplatin on native RU as well as converted RR cells derived from MCF7. As shown in Figure 5E, the IC50 of converted RR cells was significantly higher than RU cells (66.9 μM versus 44.0 μM, *p* < 0.05).

### Converted RR cells are biochemically similar to RR cells

**Figure 6:**
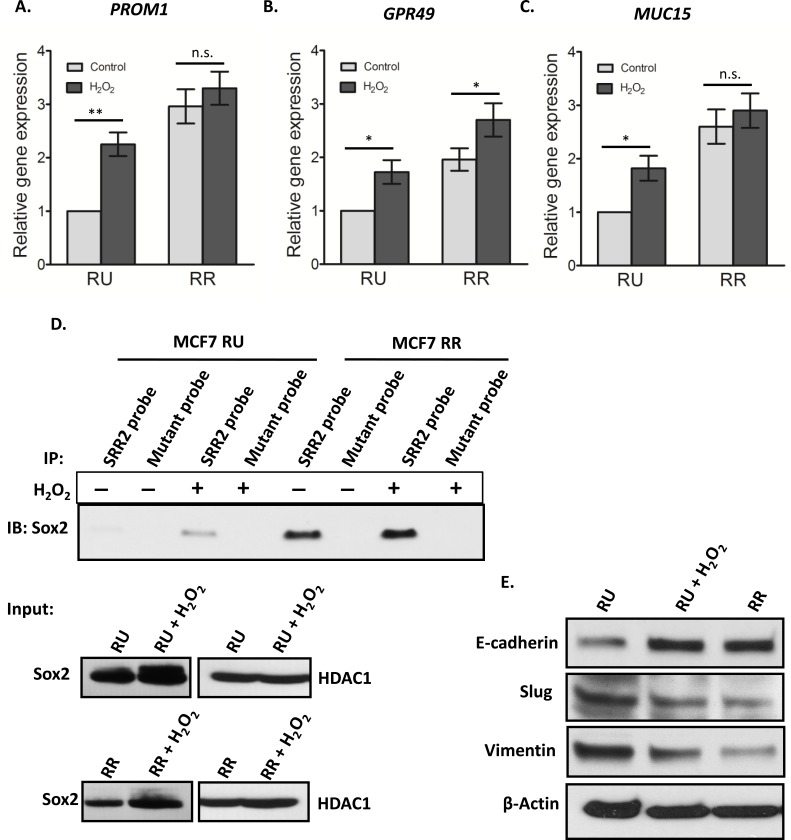
Converted MCF7 RR cells biochemically similar to RR cells RU and RR cells derived from MCF7 were exposed to 0.5 mM H_2_O_2_ for 6 hours in complete culture media and further cultured for 3 days in fresh media. The mRNA expression levels of *PROM1* **A**., *GPR49* **B**. and *MUC15* **C**. were measured by qRT-PCR by using specific primers. The data showed the significant increase in the mRNA expression of all three genes upon H_2_O_2_ treatment in the RU cells. RR cells did not show any significant change in gene expressions except in case of *GPR49*. **D**. Cell lysates derived from the nuclear fraction of H_2_O_2_ treated RU and RR cells were incubated with the biotinylated SRR2 probe. A similar amount of lysate proteins derived from treated and untreated RU and RR cells was used. The SRR2 probe-protein complexes were then captured by streptavidin beads. By western blots, Sox2 was prominently detectable in H_2_O_2_ treated RU cells contrary to very low to no detection in untreated RU cells. RR cells did not show any noticeable change in Sox2 binding to SRR2. Mutation of the SRR2 completely abrogated the binding of Sox2 to the probe (top panel). The western blot of input samples were evaluated for the quality of lysates of nuclear fractions by using an anti-Sox2 antibody. HDAC-1 was used as loading control (bottom panel). **E**. Cell lysates of H_2_O_2_ treated and untreated cells were analyzed by western blot. Protein level of E-cadherin was prominently increased, and Slug and vimentin were decreased after H_2_O_2_ treatment in RU cells in comparison to that of untreated RU cells. RR cells showed similar pattern as converted RR cells.

We then asked if converted RR cells are biochemically similar to native RR cells. We previously reported that the expression of several cancer stemness-related genes such as *PROM1 (CD133)*, *GPR49 (Lgr5)* and *MUC15* was significantly higher in RR cells as compared to RU cells derived from BC cell lines as well as primary tumor samples [29]. Thus, we compared the expression of these signature 3 genes between converted RR cells and native RU cells. As shown in Figure 6A-6C, converted RR cells had significantly higher expressions (∼2 fold) in *PROM1*, *GPR49* and *MUC15* as compared to the native RU cells (*p* < 0.05).

To further evaluate the changes in biochemical properties of Sox2, we evaluated the Sox2 binding to the SRR2 reporter. As shown by western blots, converted RR cells showed substantially higher Sox2 binding to the SRR2 as compared to native RU (Figure 6D). RR cells did not show any appreciable change in the Sox2 binding to SRR2 reporter upon H_2_O_2_ exposure. Western blot experiments showed that the total Sox2 protein level was increased in both converted RR cells and native RR cells treated with H_2_O_2_. In parallel with the previously published results [28], which showed that enforced expression of Sox2 in native RU cells did not induce GFP or luciferase expression, we found that transfection of Sox2 into RU cells did not result in an increase in Sox2-probe binding (not shown).

We also asked if the observed RU/RR conversion in response to oxidative stress is linked to epithelial mesenchymal transition (EMT), a process that is also known to be associated with an acquisition of cancer stemness [35]. As shown in Figure 6E, converted RR cells derived from MCF7 showed an appreciable increase in the protein level of E-cadherin and a corresponding decrease in the level of vimentin and Slug, a well-known negative regulator of E-cadherin (Figure 6E). This pattern of protein expression was similar to that of native RR cells.

### H_2_O_2_-induced RR conversion in a mouse xenograft model

**Figure 7:**
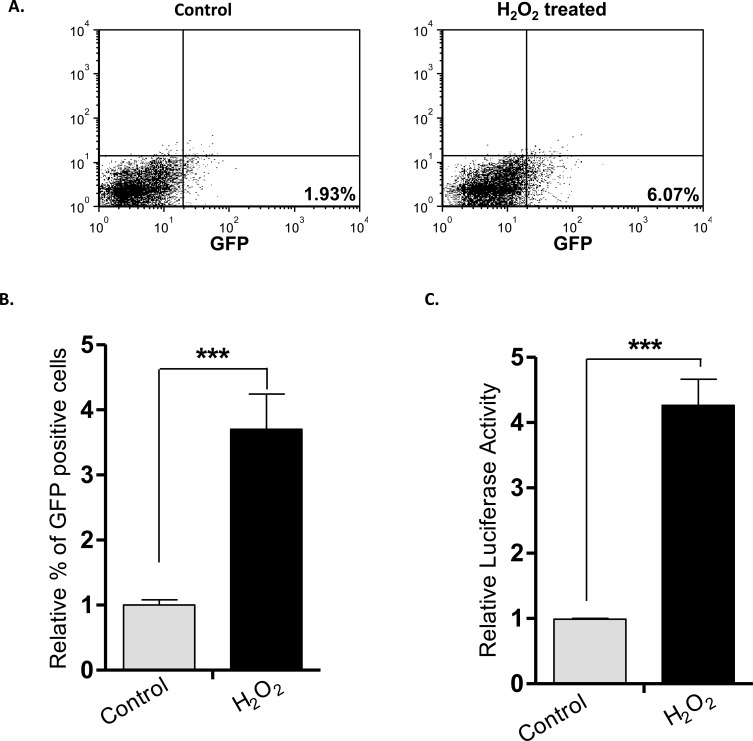
H_2_O_2_-induced RU/RR conversion *in-vivo* RU cells derived from MCF7 were injected subcutaneously in both flanks of SCID/Beige female mice. When tumor size reached 0.5mm, H_2_O_2_ (0.1mM) was injected intra-tumorally into one flank whereas xenograft on the opposite flank was left untreated. After 24 hours of H_2_O_2_ injection, tumors were subjected to flow cytometry and luciferase activity. (**A**. Flow cytometry results of a representative mouse are illustrated, and we observed a higher proportion of GFP-positive tumor cells in the xenograft treated with H_2_O_2_, as compared to the untreated xenograft on the opposite flank. (**B**. & **C**.) The average relative % of GFP-positive cells and luciferase activity of the 5 H_2_O_2_-treated xenografts were significantly different from those of their untreated counterparts (***p < 0.001).

To address the question of whether the H_2_O_2_-induced RU/RR conversion occurred in an *in-vivo* experimental system, we employed a mouse xenograft model. Thus, we injected RU cells derived from MCF7 cells into the mammary fat pad of mice (n=5). As the tumor reached 0.5 mm in the greatest dimension, H_2_O_2_ (0.1 mM) was injected intra-tumorally. Animals were euthanized 24 hours after the injection and the harvested tumor cells were examined for GFP and luciferase expression. As illustrated in Figure 7A-7B, H_2_O_2_-treated xenograft tumor cells showed a significant increase in GFP-positive cells (*p* < 0.001) and luciferase activity (*p* < 0.001), compared to untreated xenograft tumor cells (Figure 7C).

### H_2_O_2_-induced RR conversion also occurred in primary patient samples

**Figure 8:**
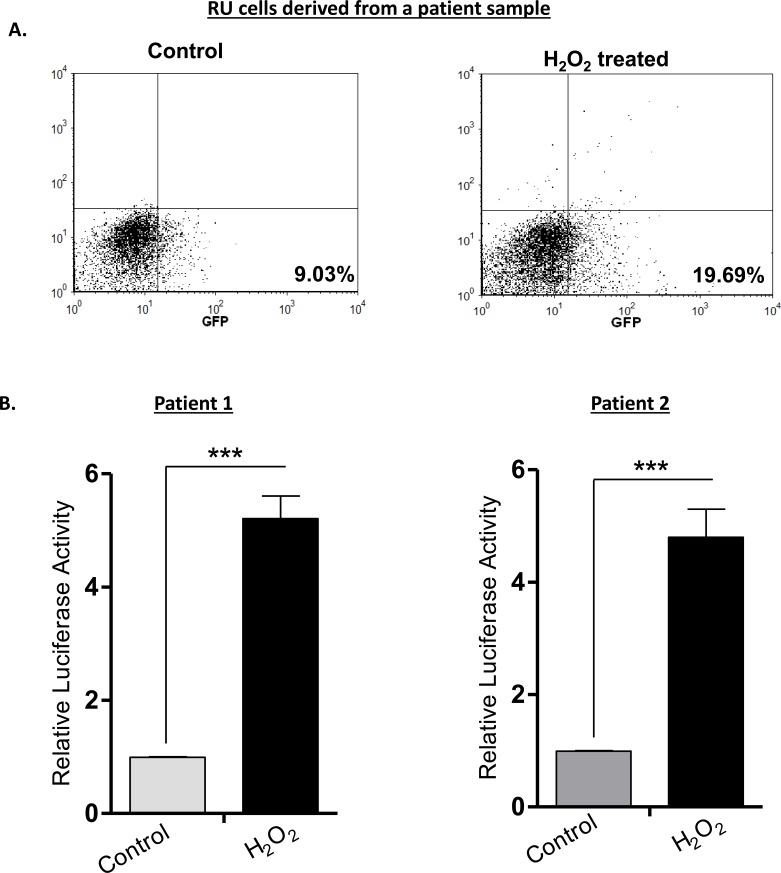
RU cells from patient samples can also convert to RR cells upon H_2_O_2_ treatment Tumor cells from 2 primary patient breast tumor samples were infected with the lentiviral SRR2 reporter and RU cells were sorted using flow cytometry. Purified RU cells were treated with H_2_O_2_ (0.5 mM) for 6 hours and analyzed for GFP and luciferase activity. **A**. Flow cytometry results of a representative sample are illustrated, and we observed an increase in the proportion of GFP-positive tumor cells after H_2_O_2_ treatment. **B**. The luciferase activity of both tumor samples treated H_2_O_2_ was significantly higher than that of the negative (untreated) controls.

Lastly, we examined if the H_2_O_2_-induced RU/RR conversion occurs in primary patient samples. We purified RU cells derived from two patient samples and treated the cells with 0.5 mM of H_2_O_2_. We then performed flow cytometry and luciferase assay 6 hours after H_2_O_2_ treatment. As illustrated in Figure 8A, flow cytometric analysis revealed an appreciable increase in GFP-positive cells (i.e. from 9% to 19%) in one patient sample. Luciferase assay in triplicate were performed for both samples, and we found a significant increase in luciferase activity in response to H_2_O_2_ in both samples (*p* < 0.001) (Figure 8B).

## DISCUSSION

Oxidative stress is related to the generation of various reactive oxygen species (ROS), such as hydroxyl radical (OH^−^), superoxide anions (O_2_^−^), and hydrogen peroxide (H_2_O_2_), all of which are produced during normal aerobic metabolism [36]. Multiple cellular mechanisms exist to minimize oxidative stress and its detrimental effects to the cells [37]. Oxidative stress and ROS are highly relevant in cancer biology. Since tumors frequently outgrow their blood supply, cancer cells are constantly subjected to glucose deprivation and hypoxia, both of which are strong inducers of ROS and oxidative stress [38, 39]. ROS and oxidative stress have been shown to increase anchorage-independent growth, motility, and cell survival in cancer [40-42]. There is also evidence that ROS and oxidative stress can increase cancer stemness [15-17]. Importantly, CSCs are known to be more tolerant to oxidative stress, which correlates well with the observation that CSCs contain a significantly lower ROS level compared to the non-stem cell population [43]. These published findings prompted us to test if RR cells, believed to be more stem-like than RU cells, are more tolerant to oxidative stress.

Our observation that RR cells were more tolerant to H_2_O_2_ than RU supports that RR cells are more stem-like than RU cells. Similar to CSCs, normal stem cells are also known to be more tolerant to oxidative damage through several mechanisms, including (a) promotion of glycolysis, (b) low oxygen consumption, (c) decreased mitochondrial mass, (d) down-regulation of ROS-producing enzymes, and (e) upregulation of ROS scavengers [7]. A recent study discovered that the CSC marker CD44, in particular the CD44v isoform, interacts and stabilizes xCT, a subunit of a glutamate-cystine transporter, to promote the uptake of cystine for the synthesis of the master antioxidant glutathione [44]. While the exact mechanism(s) underlying the oxidative stress tolerance of RR cells needs to be further investigated, our studies have provided evidence that Sox2 plays a direct role, since siRNA knockdown of this protein significantly decreased the tolerance of RR cells to the level of RU cells. At the same time, siRNA knockdown of Sox2 in RU cells, which express a similar Sox2 protein level as RR cells [28], did not result in any significant change to their tolerance to H_2_O_2_. Taken together, it is highly likely that the differential sensitivity to H_2_O_2_ between RR and RU cells is directly attributed to their differential Sox2 transcriptional status, the defining criterion used to separate RR from RU cells.

One key observation of this study is related to the H_2_O_2_-induced conversion from RU cells to RR cells (i.e. converted RR cells). As indicated in our results, this conversion is not restricted to BC cell lines used *in-vitro*, as we found a similar phenomenon in BC cell lines *in-vivo* as well as primary BC tumor cells. Considering the concept that RR cells are more stem-like than RU cells, this observation suggests that H_2_O_2_ can induce the acquisition of cancer stemness, a concept that have been previously suggested. For instance, it has been found that irradiation can enrich cancer stem-like cells in MCF7 [45, 46]. Furthermore, compared to tumor samples at diagnosis, post-chemotherapy tumor samples often contained a higher proportion of cancer stem-like cells that carry enhanced mammosphere forming efficiency and metastatic potential [4, 47]. *In vitro* experiments also have shown that hypoxia, which is known to increase oxidative stress, can induce the expression of CSC markers in BC [48].

We have previously shown that Sox2 binds to largely exclusive genes sets between RU and RR cells; as a result, there are substantial differences in their gene expression patterns [29]. In the same study, we also have highlighted the differential expression of three Sox2 downstream target genes, including *PROM1* (encoding CD133), *GPR49* (encoding Lgr5) and *MUC15*, all of which are higher expressed in RR cells. These findings correlate with our observation that Sox2 binds to SRR2 in RR cells but not RU cells [28]. In the current study, we found that Sox2 in converted RR cells can bind to SRR2. Correlating with this finding, converted RR cells adopted the gene expression pattern of the three Sox2 downstream target genes of native RR cells. Specifically, the expression level of *PROM1*, *GRP49* and *MUC15* were all up-regulated in converted RR cells. Taken together, our observations support the concept that the H_2_O_2_-induced RU/RR conversion involves the re-activation of the Sox2 transcriptional activity and a genuine biochemical re-programming, leading to a stem-like gene expression pattern. The significance of these findings can be better appreciated when the functions of these Sox2 downstream target genes are considered. The use of both CD133 and Lgr5 as surrogate markers for CSCs in BC and other cancer types has been demonstrated previously [49-51]. Lgr5 is a member of the G-protein-coupled receptor family of proteins, a target of Wnt signaling, and it is a marker of stem cells in various adult organs in humans [52]. CD133, a member of the prominin family, is expressed in normal progenitor cells as well as CSCs [53]. The role of CD133 in a wide range of cellular processes including self-renewal, tumorigenesis, metastasis, chemo-resistance, autophagy and apoptosis has been recently reviewed [54]. Regarding MUC15, it was recently described to be a Sox2 downstream target and a CSC marker in estrogen receptor-positive BC cell lines and primary tumors [29]. MUC15, a highly glycosylated extracellular protein, has been found to be elevated in the tumor initiating cell populations in colon cancer, hepatocellular carcinoma and papillary thyroid carcinoma [55-58].

EMT is a process by which cancer cells can acquire features of stemness. In this regard, it has been shown that EMT can be induced by a variety of stimuli including H_2_O_2_. For instance, *in vitro* studies using treatment of human mesothelioma cells using H_2_O_2_-induced EMT, as evidenced by the increased expression of vimentin, slug and twist1, as well as the decreased expression of E-cadherin [15]. Furthermore, ectopic expression of either Twist or Snail, both of which are key regulators of EMT, promotes the expression of CSC markers and mammosphere formation in human mammary epithelial cells [35]. With this background, we asked if our observed RU/RR conversion is related to EMT. Based on the expression pattern of E-cadherin, slug and vimentin, we concluded that the RU/RR conversion correlates with mesenchymal epithelial transition (MET) rather than EMT. This finding is somewhat surprising to us, but it is not completely contradictory to the literature. Specifically, during the reprogramming of mouse fibroblasts to iPS cells, it has been reported that Sox2 (partnered with Oct3/4) up-regulates E-cadherin by suppressing the expression of its negative regulators, Snail and Klf4 [59]. Moreover, inhibition of E-cadherin during the iPS transformation was found to decrease the yield of iPS cells [60]. While further studies need to be done in clarifying the role of EMT or MET in the context of RU/RR conversion, it is tempting to speculate that RU/RR conversion may involve some of the biochemical modulations implicated in the induction of iPS cells. In support of this concept, Sox2 is indeed one of the 4 important iPS factors initially reported by *Takahashi et. al.* [19].

The concept of “tumor cell plasticity” has been recently reviewed [61]. Sox2 has been implicated in the process in which non-stem cells convert into CSCs. In gliobastoma, Sox2 has been reported to regulate the expression of key genes involved in cancer stemness; Sox2 knockdown abolished de-differentiation and the acquisition of CSC phenotype [62]. In colorectal cancer, introduction of a set of defined factors (Oct3/4, Sox2 and Klf4) showed an enhancement in CSC properties such as sphere formation capability, expression of CSC marker genes, chemo-drug resistance, and tumorigenicity [63]. Sox2 also has been reported to be aberrantly expressed in pancreatic cancer and contributes to cell proliferation and stemness/dedifferentiation through the regulation of a set of genes controlling G1/S transition [64]. In BC, it was found that over-expression of Sox2 increased mammosphere formation whereas knockdown of Sox2 delayed tumour formation in xenograft tumour initiation models [65]. Recently, H3K9 demethylation and Sox2 gene expression have been reported to be essential for the elevation of self-renewal capability of differentiated melanoma cells [66].

From the technical point of view, we believe that our findings related to the RU/RR conversion are highly important and relevant to the future studies of CSCs, particularly with respect to the acquisition of cancer stemness in response to various stimuli. In contrast with the conventional EMT experiments, detection of increased stemness can be readily detectable by using flow cytometry in a quantitative fashion. Since the Sox2 reporter also contains the luciferase gene, localization and tracking of stem-like cells (i.e. RR cells) in animal xenograft studies can be achieved by *ex-vivo* imaging. With this model, one can readily isolate converted RR cells and delineate the molecular mechanisms that are responsible for the conversion and acquisition of cancer stemness.

In conclusion, our findings have further supported the biological significance of the RU/RR dichotomy and the concept that RR cells are more stem-like than RU cells. We believe that the H_2_O_2_-induced RU/RR conversion is a valuable model to study stress-induced acquisition of cancer stemness.

## MATERIALS AND METHODS

### Cell lines, cell culture, and reagents

Parental ER + breast cancer cell lines MCF7 and ZR751were purchased from American Type Culture Collection (ATCC, Rockville, USA). The RU and RR cell lines are derived as previously described [28]. The RU and RR cells have been purified based on GFP expression from the parental cells stably infected with the Sox2 reporter (SRR2). Since the SRR2 reporter carries dual reporter genes encoding luciferase and green fluorescence protein (GFP), RR cells but not RU cells stably show luciferase activity and GFP expression over time [28]. RU and RR cells are maintained and culture separately for our studies, and keep their distinct phenotypes [28]. All the above mentioned cell lines were maintained in high glucose Dulbecco's Modified Eagle Medium (DMEM) (Life Technologies, Grand Island, NY, USA) supplemented with 10% fetal bovine serum (FBS) (Life Technologies, Grand Island, NY, USA) and 10 μg/ml puromycin (in-case of SRR2 stable cells) (Life Technologies, Grand Island, NY, USA). H_2_O_2_ (Fisher scientific, ON, Canada), N-acetyl-L-cysteine (NAC) and L-buthionine-S,R-sulfoximine (BSO) (Sigma-Aldrich Canada, Oakville, ON, Canada) were freshly prepared.

### H_2_O_2_, NAC and BSO treatment

About 5 × 10^5^ cells were seeded into a 6-well plate. Cells were treated with 0.5 mM to 5 mM H_2_O_2_ in serum free media for 1, 2 and 4 hours. These cells were subjected to cell proliferation assay, luciferase reporter assay and flow cytometry for assessment of GFP expression. To assess the phenotypic and biochemical changes after H_2_O_2_ treatment, cells were exposed to 0.5 mM H_2_O_2_ in DMEM (Life Technologies, Grand Island, NY, USA) supplemented with 10% FBS (Life technologies, Grand Island, NY, USA) for 6 hours and further cultured in fresh media supplemented with FBS under an atmosphere of 5% CO_2_ at 37°C for 3 days. Cells were then harvested and subjected to cell proliferation assay, mammosphere formation assay, cisplatin as well as H_2_O_2_ resistance assay and biochemical characterizations. To assess if the glutathione modulation affects the H_2_O_2_ effect, cells were initially treated with 100 μM of BSO for 48 hours and 10 mM NAC for 24 hours, followed by exposure to 5 mM H_2_O_2_ for 2 hours in serum free media. These cells were subjected to luciferase assay and flow cytometry for the evaluation of GFP expression.

### Nuclear cytoplasmic fractionation

Nuclear and cytoplasmic proteins of cells were extracted using the NE-PER Protein Extraction Kit (#78833, Thermo Scientific, USA) according to the manufacturer's protocol. For western blotting analyses, histone deacetylase 1 (HDAC1, Santa Cruz biotechnology Inc. USA) were used as marker for nuclear fraction.

### SRR2-pull down for Sox2 binding

Biotinylated SRR2 wild type and mutant probes were synthesized from Integrated DNA Technologies, USA. SRR2 sequence is 5′-AAGAATTTCCCGGGCTCGGGCAGCCATTGTG ATGCATATAGGATTATTCACGTGGTAATG-3′ in which the Sox2 consensus sequence is underlined. An equal amount of nuclear protein (300 μg) was incubated with 3 pmole of either mutant or wild-type SRR2 probe for 30 min at room temperature. Then 75 μl streptavidin beads were added and the samples were incubated overnight by rotation at 4°C. The beads were then washed 3 times with cold PBS. The bound proteins were eluted by boiling at 95°C for 5 min with 20 μL of SDS protein buffer and then processed for western blotting.

### Western blotting

Western blot analyses were performed as previously described [29]. All antibodies were diluted in 5% bovine serum albumin (BSA) in Tris buffered saline and 0.1% Tween-20 (TBST): anti-Sox2 (1:1000, Cat. #3579), anti-Vimentin (1:1000, Cat. #5741), anti-Slug (1:500, Cat. #9585), and anti-E-cadherin (1:1000, Cat. #3195) were purchased from Cell Signaling Technologies, Danvers, MA, USA. Anti-β-actin (1:5000, # 47778) was purchased from Santa Cruz Biotechnology, USA. The expression of beta-actin served as the loading control for all western blots.

### siRNA knockdown of Sox2

Sox2 siRNAs (SMARTpool: ON-TARGETplus Sox2 siRNA, Dharmacon, Fisher Scientific, ON, Canada) or scrambled (Scr) siRNAs (ON-TARGETplus Non-targeting Pool, #477C20, Dharmacon, Fischer Scientific, ON, Canada) at 40 pmol per rxn (20 nM final concentration) and 5 μL of Lipofectamine RNAiMAX (Life Technologies, Grand Island, NY, USA) were added to 0.5 mL of OptiMEM media (Life Technologies, Grand Island, NY, USA) and reverse transfected to 0.8×10^6^ cells in normal culture medium in a 6-well plate format. Cells were incubated with siRNAs for 48 hours before harvesting.

### Cell proliferation assay, luciferase reporter assay and flow cytometry

Cell proliferation was determined using the 3-(4,5-dimethylthiazol-2-yl)-5-(3-carboxymethoxyphenyl)-2-(4-sulfophenyl)-2H-tetrazolium, inner salt (MTS) assay (#G3580, Promega, Madison, WI, USA) according to the manufacturer's instructions. Luciferase reporter assay was performed using luciferase assay system kit (#E4530, Promega, Corporation, Madison, USA) according to the manufacturer's protocol, plated on Costar white polystyrene opaque 96-well plates (#3912, Corning, NY, USA) and analyzed on the FLUOstar Omega multi-mode microplate reader (BMG Labtech, Ortenburg, Germany). Flow cytometry analyses were performed as previously described [28].

### Limiting dilution and mammosphere formation assay

Mammospheres were seeded and cultured as previously described [67]. Briefly, cells were trypsinized and passed through a 40 μm cell strainer (BD, Franklin Lakes, New Jersey, USA) and seeded into ultra-low adherent plates (Corning, NY, USA) in Mammocult media (StemCell Technologies, Vancouver, BC, Canada) as per manufacturer's instructions. Mammosphere larger than 60 μm were counted 5-7 days after seeding. Limiting dilution assay has been used as a gold standard for the assessment of cancer stem cells [33, 34]. To perform these experiments, cells were seeded in 96-well low-adherent plate (Corning, NY, USA) at 10 limiting dilutions ranging from 1 to 400 cells. Each dilution had 6 replicates, and each well was scored for presence or absence of mammosphere after 5-7 days. Data were analyzed using the Extreme Limiting Dilition Analysis (ELDA) software for three independent experiments [68].

### RNA extraction, cDNA synthesis, quantitative reverse transcription PCR (q-RT-PCR)

Total RNA extraction was performed with the Qiagen RNeasy Kit (Qiagen, Canada) according to the manufacturer's protocol: 1 μg of RNA was reverse transcribed using oligo-dT and superscript II (Life Technologies, Grand Island, NY, USA) according to the manufacturer's protocol. 1 μL of the resulting cDNA mixture was added to the Platinum SYBR Green qPCR SuperMix-UDG with Rox (Life Technologies, Grand Island, NY, USA) and amplified with target gene-specific primers. Please see Additional file 2: Table S1 for list of primer sequences. All genes of interest are normalized to glyceraldehyde-3-phosphate dehydrogenase (GAPDH) transcript expression levels.

### SCID mouse xenograft studies

Five SCID/Beige mice purchased from Taconic (Hudson, NY) were kept virus- and antigen-free and housed in the Alberta Institute for Viral Immunology biocontainment facility at University of Alberta. Our experimental protocols had been reviewed and approved by the institutional Animal Welfare Committee, and animals care was provided in accordance with the 1993 guidelines of the Canadian Council on Animal Care. RU cells derived from MCF7 were re-suspended in 1:1 Matrigel/PBS. All mice at 8-10 weeks old received two subcutaneous injections of 5×10^6^ RU cells in 200 uL of 1:1 Matrigel/PBS solution one on either flank side. A slow release pellet of estradiol (1.7 mg, Innovative Research) was implanted subcutaneously at the nape of the neck to promote tumor growth. When tumor size reached ≥5mm, H_2_O_2_ (0.1mM) was administered by intra-tumoral injection (50 uL) on one flank, while the tumor on the opposite flank was untreated. After the H_2_O_2_ injection (24 hours), animals were euthanized and tumor cells were dissociated using the Macs tumor dissociation kit (Miltenyi Biotec, Auburn, CA) as per the manufacturer's protocol. Tumors cells were then evaluated for SRR2 reporter activity by using flow cytometry and Luciferase activity.

### Patient samples and experimental manipulations

Two primary patient tumors were processed using a protocol described previously [29]. Briefly, BC cells were harvested by using the Cancer Cell Isolation Kit (Panomics Solutions, Affymetrix, Santa Clara, CA) as per the manufacturer's protocol. Isolated tumor cells were infected with our modified lentiviral Sox2 GFP-RFP dual-color reporter, SRR2-mCMV-GFP-EF1-RFP. RU cells (RFP+/GFP-) were sorted using flow cytometry. Sorted RU cells were cultured for 24 hours in RPMI media followed by treatment with H_2_O_2_ (0.5 mM). After 6 hours of H_2_O_2_ treatment, tumors cells were analyzed for GFP expression and Luciferase activity.

### Statistical analyses

Data are expressed as mean ± standard errors. IC50 was calculated by Graphpad Prism (La Jolla, CA). Paired Student's T-tests were used for statistical analyses of experiments throughout, where *p* < 0.05 is denoted by *, *p* < 0.01 is denoted by **, and *p* < 0.001 is denoted by ***. All graphs represent the average of at least 3 independent experiments with triplicates.

## SUPPLEMENTARY MATERIAL TABLE AND FIGURE



## References

[1] Sarrio D, Franklin CK, Mackay A, Reis-Filho JS, Isacke CM (2012). Epithelial and mesenchymal subpopulations within normal basal breast cell lines exhibit distinct stem cell/progenitor properties. Stem Cells.

[2] Al-Hajj M, Wicha MS, Benito-Hernandez A, Morrison SJ, Clarke MF (2003). Prospective identification of tumorigenic breast cancer cells. Proc Natl Acad Sci U S A.

[3] Sheridan C, Kishimoto H, Fuchs RK, Mehrotra S, Bhat-Nakshatri P, Turner CH, Goulet R, Badve S, Nakshatri H (2006). CD44+/CD24- breast cancer cells exhibit enhanced invasive properties: an early step necessary for metastasis. Breast Cancer Res.

[4] Li X, Lewis MT, Huang J, Gutierrez C, Osborne CK, Wu MF, Hilsenbeck SG, Pavlick A, Zhang X, Chamness GC, Wong H, Rosen J, Chang JC (2008). Intrinsic resistance of tumorigenic breast cancer cells to chemotherapy. J Natl Cancer Inst.

[5] Thiery JP, Acloque H, Huang RY, Nieto MA (2009). Epithelial-mesenchymal transitions in development and disease. Cell.

[6] Fillmore CM, Kuperwasser C (2008). Human breast cancer cell lines contain stem-like cells that self-renew, give rise to phenotypically diverse progeny and survive chemotherapy. Breast Cancer Res.

[7] Liu J, Wang Z (2015). Increased Oxidative Stress as a Selective Anticancer Therapy. Oxidative Medicine and Cellular Longevity.

[8] Cardiff RD, Couto S, Bolon B (2011). Three interrelated themes in current breast cancer research: gene addiction, phenotypic plasticity, and cancer stem cells. Breast Cancer Res.

[9] Roesch A, Fukunaga-Kalabis M, Schmidt EC, Zabierowski SE, Brafford PA, Vultur A, Basu D, Gimotty P, Vogt T, Herlyn M (2010). A temporarily distinct subpopulation of slow-cycling melanoma cells is required for continuous tumor growth. Cell.

[10] Schwitalla S, Fingerle AA, Cammareri P, Nebelsiek T, Goktuna SI, Ziegler PK, Canli O, Heijmans J, Huels DJ, Moreaux G, Rupec RA, Gerhard M, Schmid R, Barker N, Clevers H, Lang R (2013). Intestinal tumorigenesis initiated by dedifferentiation and acquisition of stem-cell-like properties. Cell.

[11] Sharma SV, Lee DY, Li B, Quinlan MP, Takahashi F, Maheswaran S, McDermott U, Azizian N, Zou L, Fischbach MA, Wong KK, Brandstetter K, Wittner B (2010). A chromatin-mediated reversible drug-tolerant state in cancer cell subpopulations. Cell.

[12] Chaffer CL, Brueckmann I, Scheel C, Kaestli AJ, Wiggins PA, Rodrigues LO, Brooks M, Reinhardt F, Su Y, Polyak K, Arendt LM, Kuperwasser C, Bierie B (2011). Normal and neoplastic nonstem cells can spontaneously convert to a stem-like state. Proc Natl Acad Sci U S A.

[13] Heddleston JM, Li Z, McLendon RE, Hjelmeland AB, Rich JN (2009). The hypoxic microenvironment maintains glioblastoma stem cells and promotes reprogramming towards a cancer stem cell phenotype. Cell Cycle.

[14] Li P, Zhou C, Xu L, Xiao H (2013). Hypoxia enhances stemness of cancer stem cells in glioblastoma: an *in vitro* study. Int J Med Sci.

[15] Kim MC, Cui FJ, Kim Y (2013). Hydrogen peroxide promotes epithelial to mesenchymal transition and stemness in human malignant mesothelioma cells. Asian Pac J Cancer Prev.

[16] Le Belle JE, Orozco NM, Paucar AA, Saxe JP, Mottahedeh J, Pyle AD, Wu H, Kornblum HI (2011). Proliferative neural stem cells have high endogenous ROS levels that regulate self-renewal and neurogenesis in a PI3K/Akt-dependant manner. Cell Stem Cell.

[17] Das B, Tsuchida R, Malkin D, Koren G, Baruchel S, Yeger H (2008). Hypoxia enhances tumor stemness by increasing the invasive and tumorigenic side population fraction. Stem Cells.

[18] Sarkar A, Hochedlinger K (2013). The sox family of transcription factors: versatile regulators of stem and progenitor cell fate. Cell Stem Cell.

[19] Takahashi K, Tanabe K, Ohnuki M, Narita M, Ichisaka T, Tomoda K, Yamanaka S (2007). Induction of pluripotent stem cells from adult human fibroblasts by defined factors. Cell.

[20] Annovazzi L, Mellai M, Caldera V, Valente G, Schiffer D (2011). SOX2 expression and amplification in gliomas and glioma cell lines. Cancer Genomics Proteomics.

[21] Chen Y, Shi L, Zhang L, Li R, Liang J, Yu W, Sun L, Yang X, Wang Y, Zhang Y, Shang Y (2008). The molecular mechanism governing the oncogenic potential of SOX2 in breast cancer. J Biol Chem.

[22] Laga AC, Zhan Q, Weishaupt C, Ma J, Frank MH, Murphy GF (2011). SOX2 and nestin expression in human melanoma: an immunohistochemical and experimental study. Exp Dermatol.

[23] Rudin CM, Durinck S, Stawiski EW, Poirier JT, Modrusan Z, Shames DS, Bergbower EA, Guan Y, Shin J, Guillory J, Rivers CS, Foo CK, Bhatt D, Stinson J, Gnad F, Haverty PM (2012). Comprehensive genomic analysis identifies SOX2 as a frequently amplified gene in small-cell lung cancer. Nat Genet.

[24] Zhang J, Chang DY, Mercado-Uribe I, Liu J (2012). Sex-determining region Y-box 2 expression predicts poor prognosis in human ovarian carcinoma. Hum Pathol.

[25] Ben-Porath I, Thomson MW, Carey VJ, Ge R, Bell GW, Regev A, Weinberg RA (2008). An embryonic stem cell-like gene expression signature in poorly differentiated aggressive human tumors. Nat Genet.

[26] Lengerke C, Fehm T, Kurth R, Neubauer H, Scheble V, Muller F, Schneider F, Petersen K, Wallwiener D, Kanz L, Fend F, Perner S, Bareiss PM, Staebler A (2011). Expression of the embryonic stem cell marker SOX2 in early-stage breast carcinoma. BMC Cancer.

[27] Wang X, Liang Y, Chen Q, Xu HM, Ge N, Luo RZ, Shao JY, He Z, Zeng YX, Kang T, Yun JP, Xie F (2012). Prognostic significance of SOX2 expression in nasopharyngeal carcinoma. Cancer Invest.

[28] Wu F, Zhang J, Wang P, Ye X, Jung K, Bone KM, Pearson JD, Ingham RJ, McMullen TP, Ma Y, Lai R (2012). Identification of two novel phenotypically distinct breast cancer cell subsets based on Sox2 transcription activity. Cell Signal.

[29] Jung K, Wang P, Gupta N, Gopal K, Wu F, Ye X, Alshareef A, Bigras G, McMullen TP, Abdulkarim BS, Lai R (2014). Profiling gene promoter occupancy of Sox2 in two phenotypically distinct breast cancer cell subsets using chromatin immunoprecipitation and genome-wide promoter microarrays. Breast Cancer Res.

[30] Aruoma OI, Halliwell B, Hoey BM, Butler J (1989). The antioxidant action of N-acetylcysteine: its reaction with hydrogen peroxide, hydroxyl radical, superoxide, and hypochlorous acid. Free Radic Biol Med.

[31] De Flora S, Izzotti A, D'Agostini F, Balansky RM (2001). Mechanisms of N-acetylcysteine in the prevention of DNA damage and cancer, with special reference to smoking-related end-points. Carcinogenesis.

[32] Meister A (1983). Selective modification of glutathione metabolism. Science.

[33] Calcagno AM, Salcido CD, Gillet JP, Wu CP, Fostel JM, Mumau MD, Gottesman MM, Varticovski L, Ambudkar SV (2010). Prolonged drug selection of breast cancer cells and enrichment of cancer stem cell characteristics. J Natl Cancer Inst.

[34] Rota LM, Lazzarino DA, Ziegler AN, LeRoith D, Wood TL (2012). Determining mammosphere-forming potential: application of the limiting dilution analysis. J Mammary Gland Biol Neoplasia.

[35] Mani SA, Guo W, Liao MJ, Eaton EN, Ayyanan A, Zhou AY, Brooks M, Reinhard F, Zhang CC, Shipitsin M, Campbell LL, Polyak K, Brisken C, Yang J, Weinberg RA (2008). The epithelial-mesenchymal transition generates cells with properties of stem cells. Cell.

[36] Simic MG, Bergtold DS, Karam LR (1989). Generation of oxy radicals in biosystems. Mutat Res.

[37] Dreher D, Junod AF (1996). Role of oxygen free radicals in cancer development. Eur J Cancer.

[38] Lee YJ, Galoforo SS, Berns CM, Chen JC, Davis BH, Sim JE, Corry PM, Spitz DR (1998). Glucose deprivation-induced cytotoxicity and alterations in mitogen-activated protein kinase activation are mediated by oxidative stress in multidrug-resistant human breast carcinoma cells. J Biol Chem.

[39] Spitz DR, Sim JE, Ridnour LA, Galoforo SS, Lee YJ (2000). Glucose deprivation-induced oxidative stress in human tumor cells. A fundamental defect in metabolism?. Ann N Y Acad Sci.

[40] Burdick AD, Davis JW, Liu KJ, Hudson LG, Shi H, Monske ML, Burchiel SW (2003). Benzo(a)pyrene quinones increase cell proliferation, generate reactive oxygen species, and transactivate the epidermal growth factor receptor in breast epithelial cells. Cancer Res.

[41] Irani K, Xia Y, Zweier JL, Sollott SJ, Der CJ, Fearon ER, Sundaresan M, Finkel T, Goldschmidt-Clermont PJ (1997). Mitogenic signaling mediated by oxidants in Ras-transformed fibroblasts. Science.

[42] Ruiz-Ramos R, Lopez-Carrillo L, Rios-Perez AD, De Vizcaya-Ruiz A, Cebrian ME (2009). Sodium arsenite induces ROS generation, DNA oxidative damage, HO-1 and c-Myc proteins, NF-kappaB activation and cell proliferation in human breast cancer MCF-7 cells. Mutat Res.

[43] Naka K, Muraguchi T, Hoshii T, Hirao A (2008). Regulation of reactive oxygen species and genomic stability in hematopoietic stem cells. Antioxid Redox Signal.

[44] Ishimoto T, Nagano O, Yae T, Tamada M, Motohara T, Oshima H, Oshima M, Ikeda T, Asaba R, Yagi H, Masuko T, Shimizu T, Ishikawa T, Kai K, Takahashi E, Imamura Y (2011). CD44 variant regulates redox status in cancer cells by stabilizing the xCT subunit of system xc(−) and thereby promotes tumor growth. Cancer Cell.

[45] Phillips TM, McBride WH, Pajonk F (2006). The response of CD24(−/low)/CD44+ breast cancer-initiating cells to radiation. J Natl Cancer Inst.

[46] Woodward WA, Chen MS, Behbod F, Alfaro MP, Buchholz TA, Rosen JM (2007). WNT/beta-catenin mediates radiation resistance of mouse mammary progenitor cells. Proc Natl Acad Sci U S A.

[47] Charafe-Jauffret E, Ginestier C, Iovino F, Wicinski J, Cervera N, Finetti P, Hur MH, Diebel ME, Monville F, Dutcher J, Brown M, Viens P, Xerri L, Bertucci F, Stassi G, Dontu G (2009). Breast cancer cell lines contain functional cancer stem cells with metastatic capacity and a distinct molecular signature. Cancer Res.

[48] van den Beucken T, Koch E, Chu K, Rupaimoole R, Prickaerts P, Adriaens M, Voncken JW, Harris AL, Buffa FM, Haider S, Starmans MH, Yao CQ, Ivan M, Ivan C, Pecot CV, Boutros PC (2014). Hypoxia promotes stem cell phenotypes and poor prognosis through epigenetic regulation of DICER. Nat Commun.

[49] Nakata S, Phillips E, Goidts V (2014). Emerging role for leucine-rich repeat-containing G-protein-coupled receptors LGR5 and LGR4 in cancer stem cells. Cancer Manag Res.

[50] Wu F, Ye X, Wang P, Jung K, Wu C, Douglas D, Kneteman N, Bigras G, Ma Y, Lai R (2013). Sox2 suppresses the invasiveness of breast cancer cells via a mechanism that is dependent on Twist1 and the status of Sox2 transcription activity. BMC Cancer.

[51] Wu Y, Wu PY (2009). CD133 as a marker for cancer stem cells: progresses and concerns. Stem Cells Dev.

[52] Sato T, Clevers H (2013). Growing self-organizing mini-guts from a single intestinal stem cell: mechanism and applications. Science.

[53] Yu X, Lin Y, Yan X, Tian Q, Li L, Lin EH (2011). CD133, Stem Cells, and Cancer Stem Cells: Myth or Reality?. Curr Colorectal Cancer Rep.

[54] Li Z (2013). CD133: a stem cell biomarker and beyond. Exp Hematol Oncol.

[55] Huang J, Che MI, Huang YT, Shyu MK, Huang YM, Wu YM, Lin WC, Huang PH, Liang JT, Lee PH, Huang MC (2009). Overexpression of MUC15 activates extracellular signal-regulated kinase 1/2 and promotes the oncogenic potential of human colon cancer cells. Carcinogenesis.

[56] Nam KH, Noh TW, Chung SH, Lee SH, Lee MK, Hong SW, Chung WY, Lee EJ, Park CS (2011). Expression of the membrane mucins MUC4 and MUC15, potential markers of malignancy and prognosis, in papillary thyroid carcinoma. Thyroid.

[57] Pallesen LT, Berglund L, Rasmussen LK, Petersen TE, Rasmussen JT (2002). Isolation and characterization of MUC15, a novel cell membrane-associated mucin. Eur J Biochem.

[58] Wang RY, Chen L, Chen HY, Hu L, Li L, Sun HY, Jiang F, Zhao J, Liu GM, Tang J, Chen CY, Yang YC, Chang YX, Liu H, Zhang J, Yang Y (2013). MUC15 inhibits dimerization of EGFR and PI3K-AKT signaling and is associated with aggressive hepatocellular carcinomas in patients. Gastroenterology.

[59] Li R, Liang J, Ni S, Zhou T, Qing X, Li H, He W, Chen J, Li F, Zhuang Q, Qin B, Xu J, Li W, Yang J, Gan Y, Qin D (2010). A mesenchymal-to-epithelial transition initiates and is required for the nuclear reprogramming of mouse fibroblasts. Cell Stem Cell.

[60] Chen T, Yuan D, Wei B, Jiang J, Kang J, Ling K, Gu Y, Li J, Xiao L, Pei G (2010). E-cadherin-mediated cell-cell contact is critical for induced pluripotent stem cell generation. Stem Cells.

[61] Friedmann-Morvinski D, Verma IM (2014). Dedifferentiation and reprogramming: origins of cancer stem cells. EMBO Rep.

[62] Berezovsky AD, Poisson LM, Cherba D, Webb CP, Transou AD, Lemke NW, Hong X, Hasselbach LA, Irtenkauf SM, Mikkelsen T, deCarvalho AC (2014). Sox2 promotes malignancy in glioblastoma by regulating plasticity and astrocytic differentiation. Neoplasia.

[63] Oshima N, Yamada Y, Nagayama S, Kawada K, Hasegawa S, Okabe H, Sakai Y, Aoi T (2014). Induction of cancer stem cell properties in colon cancer cells by defined factors. PLoS One.

[64] Herreros-Villanueva M, Zhang JS, Koenig A, Abel EV, Smyrk TC, Bamlet WR, de Narvajas AA, Gomez TS, Simeone DM, Bujanda L, Billadeau DD (2013). SOX2 promotes dedifferentiation and imparts stem cell-like features to pancreatic cancer cells. Oncogenesis.

[65] Leis O, Eguiara A, Lopez-Arribillaga E, Alberdi MJ, Hernandez-Garcia S, Elorriaga K, Pandiella A, Rezola R, Martin AG (2012). Sox2 expression in breast tumours and activation in breast cancer stem cells. Oncogene.

[66] Tan Y, Tajik A, Chen J, Jia Q, Chowdhury F, Wang L, Chen J, Zhang S, Hong Y, Yi H, Wu DC, Zhang Y, Wei F, Poh YC, Seong J, Singh R (2014). Matrix softness regulates plasticity of tumour-repopulating cells via H3K9 demethylation and Sox2 expression. Nat Commun.

[67] Ye X, Wu F, Wu C, Wang P, Jung K, Gopal K, Ma Y, Li L, Lai R (2014). beta-Catenin, a Sox2 binding partner, regulates the DNA binding and transcriptional activity of Sox2 in breast cancer cells. Cell Signal.

[68] Hu Y, Smyth GK (2009). ELDA: extreme limiting dilution analysis for comparing depleted and enriched populations in stem cell and other assays. J Immunol Methods.

