# Supplementary material for: Oxidative stress induces the acquisition of cancer stem-like phenotype in breast cancer detectable by using a Sox2 regulatory region-2 (SRR2) reporter
Source: Oncotarget. 2015 Dec 16;7(3):3111–27. doi: 10.18632/oncotarget.6630 (PMC4823094; doi:10.18632/oncotarget.6630)
Supplement: Supplementary file 1 [file oncotarget-07-3111-s001.pdf]

Oxidative stress induces the acquisition of cancer stem-like phenotype in breast cancer detectable by using a Sox2 regulatory region-2 (SRR2) reporter

Supplementary Material

Supplementary Table 1: Primer sequences used for qRT-PCR.

GAPDH - F	GTCTCCTCTGACTTCAACAGCG	GAPDH - R	ACCACCCTGTTGCTGTAGCCAA
PROM1 - F	AGTCGGAAACTGGCAGATAGC	PROM1 - R	GGTAGTGTTGTACTGGGCCAAT
GPR49 - F	CTCCCAGGTCTGGTGTGTTG	GPR49 - R	GAGGTCTAGGTAGGAGGTGAAG
MUC15 - F	TATTCACTTCTATCGGGGAGCC	MUC15 - R	GGGAATGACTCGCCTTGAGAT

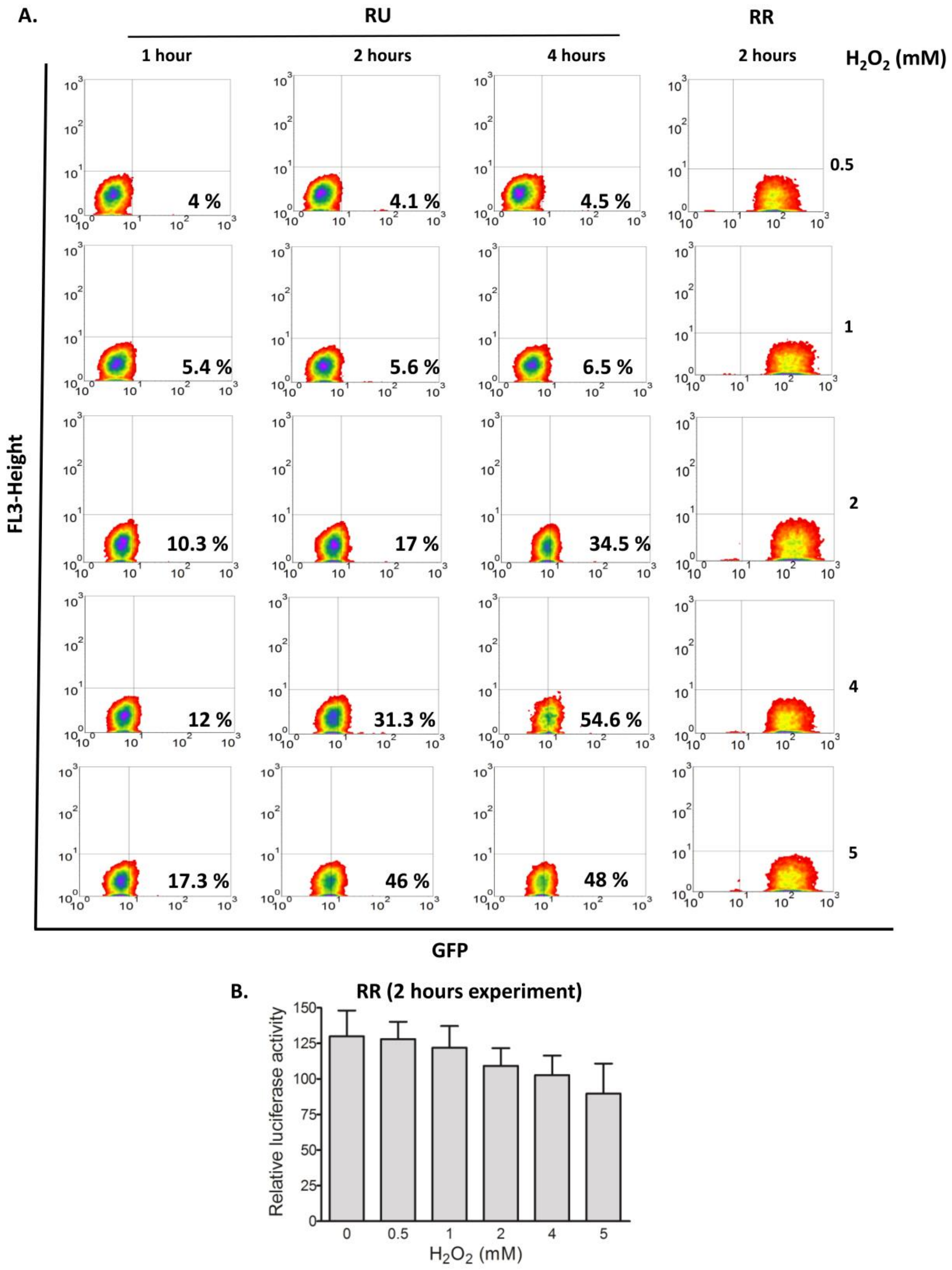

Figure S1. RU cells derived from MCF7 converted to RR cells (GFP positive) upon H_2O_2 treatment

(A) RU and RR cells were exposed to varying doses of H_2O_2 for different time points. Flow cytometry was used to assess the expression of GFP in the viable cell populations. Data is expressed relative to untreated negative control cells and the values represent the GFP positive cells. Addition of H_2O_2 to RU cells increased the proportion of GFP-positive cells in a time- and dose-dependent fashion. There was no any significant change in RR cells in the similar experimental conditions. **(B)** RR cells were exposed to varying doses of H_2O_2 for 2 hours and subjected to luciferase assay at the end of experiment. Data is expressed as luciferase activity relative to untreated negative control. There was no any significant change in luciferase activity in RR cells.
